# A New Power Dissipation Model and Its Analytic Formulation for Electric-Field-Driven Water Dissociation in the Cationic/Anionic Bipolar Polymer Membrane Junctions

**DOI:** 10.3390/membranes16030094

**Published:** 2026-03-02

**Authors:** Mohamed Fadel Anass Ma-el-ainine, Rachid Boukhili, Oumarou Savadogo

**Affiliations:** 1 Laboratory of New Materials for Energy and Electrochemistry, Polytechnique-Montréal, Montréal, QC H3T 1J4, Canada; mohamed-fadel-anass.ma-el-ainine@etud.polymtl.ca; 2 Research Centre for High Performance Polymer and Composite Systems (CREPEC), Mechanical Engineering Department, Polytechnique Montréal, Montreal, QC H3T 1J4, Canada; rachid.boukhili@polymtl.ca

**Keywords:** bipolar membranes, water dissociation, electric field, autoprotolysis, power dissipation, nanometric junction, water dissociation rate constant, new formalism, current–voltage curves

## Abstract

Bipolar Polymer Membranes (BPMs) enable the creation of large, stable pH gradients by driving water dissociation (WD) at the cation/anion junction under reverse bias, a process central to electrodialysis, CO_2_ capture, and emerging acid–alkaline water electrolysis. Yet despite decades of study, the mechanism by which intense interfacial electric fields accelerate WD remains debated and is often modeled with ad hoc assumptions. In this study, we present a power dissipation model in which minority ions from water autoprotolysis act as carriers that continuously dissipate field-supplied power in the hydrated nanometric junction. This dissipative input increases the local probability of heterolytic O–H bond cleavage and analytically leads to a quadratic dependence of the dissociation rate constant on the field. Without adjustable parameters, the model reproduces the required orders of magnitude for the enhancement ratio *k_d_*(*E*)/*k_d_*(0), where *k_d_*(*E*) is the field-enhanced water dissociation rate constant and *k_d_*(0) is its zero-field value across typical BPM fields, and yields a quadratic current–voltage junction law. A proof-of-principle measurement on a commercial Fumasep^®^ FBM bipolar membrane confirms the quadratic current–voltage trend, supporting a power-dissipation-driven water dissociation mechanism and providing a concise, falsifiable baseline for future studies.

## 1. Introduction

Bipolar Polymer Membranes (BPMs) are a unique class of polymer ion-exchange membranes (IEMs) [1], characterized by a laminated structure consisting of a cation-exchange membrane (CEM) on an anion-exchange membrane (AEM) [2]. This configuration enables BPMs to promote water dissociation (WD) at the junction of the two layers under reverse polarization [3], thereby sustaining large pH gradients across the membrane by generating hydronium (H_3_O^+^) and hydroxide (OH^−^) ions [4]. Originally developed for electrodialysis (ED) to produce acids and bases [5,6], BPMs have recently garnered substantial interest in advanced electrochemical applications [7], including brine valorization [8], CO_2_ capture [9], fuel cells [10], and particularly water electrolysis for hydrogen production [11,12]. Until now, the detailed mechanism of WD in BPMs—especially under intense interfacial electric fields—remained poorly understood. Under reverse bias, counter-ions migrate away from the CEM/AEM interface, creating a narrow space-charge region where an electric field exceeding 10^8^ V·m^−1^ is established [13]. This field accelerates the dissociation of water molecules at the interface into H_3_O^+^ and OH^−^ ions [8], which then migrate through the CEM and AEM, respectively. Using mass conservation, the flux of H_3_O^+^ and OH^−^ out from the junction cannot exceed their generation rate. Thus, the maximum WD current density in a BPM junction is given via the following equation [5,14]:

(1) $$J=F\times c_{H2O,j}\times k_{d}\times \delta ,$$

where the parameters J, F, $k_{d}$, $c_{H2O,j}$, and δ are the maximum WD current density in (A·m^−2^), Faraday constant (96,485 C·mol^−1^), water dissociation rate constant in (s^−1^), water concentration at the interface in (mol·m^−3^), and thickness of the BPM junction in (m), respectively. Equation (1) explicitly assumes a reaction-limited WD with negligible recombination inside the junction, which is plausible for abrupt BPM junctions, where the transition region is a few nanometers [1]. Thus, the nascent H_3_O^+^ and OH^−^ are rapidly extracted into the CEM and AEM. In homogeneous BPMs with a uniform functional-group distribution [15], the junction thickness does not exceed 1 nm [1,16]. In the absence of an external field (*E* = 0), the calculated water dissociation rate constant at 25 °C is $k_{d}\left(0\right)$ = 2.5 × 10^−5^ s^−1^ [17]. Assuming this nanometric junction is fully hydrated with pure water ($c_{H2O,j}$ = 55.5 mol·L^−1^) [5], the predicted current density is only ~1.4 × 10^−4^ A·m^−2^ [5]. However, experimentally observed BPM current densities can exceed 1000 A·m^−2^ [5], implying an enhancement of $k_{d}$ by at least 7 × 10^6^ times the thermal value $k_{d}\left(0\right)$ at 25 °C. This striking discrepancy is at the heart of a longstanding theoretical challenge. Existing models—such as the Second Wien Effect (SWE) and catalytic protonation–deprotonation mechanisms—do not consistently explain this increase. Consequently, new theoretical frameworks are needed to predict and optimize BPM performance. In this context, we propose a power dissipation-based model in which WD is driven by field-supplied power to autoprotolysis ions in water. The model establishes a quadratic dependence of $k_{d}$ on the field E, which follows a quadratic current–voltage junction law. This manuscript is organized as follows: Section 2 reviews the state-of-the-art theoretical models of water dissociation in bipolar membranes. Section 3 details the theoretical development of our power dissipation model, along with its mathematical relations and associated theoretical results. Section 4, Section 5, and Section 6 present the experimental methods, results and discussion, and conclusion, respectively.

## 2. State-of-the-Art Models for Water Dissociation (WD) Enhancement in BPM Junctions

Understanding the sharp increase in the WD rate in BPMs has been a longstanding challenge. Several theoretical models have been proposed, two of which dominate current discussions: the SWE and the catalytic protonation–deprotonation mechanism involving membrane functional groups. Despite their respective limitations, these models are widely used in BPM modeling [3,18,19,20]. We briefly summarize their scope and limitations, together with alternative approaches.

### 2.1. Second Wien Effect (SWE)

The Second Wien Effect (SWE) was the first mechanism proposed to account for the increase in WD under an electric field [21,22]. Based on Onsager’s theory [23], the effect describes how a strong electric field enhances the dissociation of ion pairs in weak electrolytes. For high electric fields (*E* > 10^8^ V·m^−1^), the ratio $\;k_{d}\left(E\right)$/$k_{d}\left(0\right)$ can be determined via the following equation [23].

(2) $$\frac{k_{d\;}\left(E\right)}{k_{d}\left(0\right)}=\sqrt{\frac{2}{\pi }}\times {\left(8b\right)}^{-\frac{3}{4}\;}\times e^{\sqrt{8b}},$$

where $b\approx 0.096\times \frac{E}{\varepsilon_{r\;}T^{2}}$, E is the field strength (V·m^−1^), $\varepsilon_{r\;}$ is the relative permittivity, and T is the temperature (K). While pure water is often treated as a weak electrolyte due to its low ionic concentration (~10^−7^ mol·L^−1^), applying SWE to water has serious limitations [24,25,26]. First, Onsager’s theory assumes pre-existing ion pairs, not neutral water molecules [27,28]. Second, the high dielectric constant of water (~80 at 25 °C) makes stable ion pair formation unlikely [22,23]. Moreover, the SWE-predicted dissociation rates remain far too low to explain the experimental results obtained using BPMs. According to Strathmann [5], it can only match observed values if $\varepsilon_{r\;}$ is reduced below 10. Krol [14] discussed the role of relative permittivity in SWE, highlighting the difficulty of determining $\varepsilon_{r}$ and its complexity when considering it as field-dependent, especially under high fields where $\varepsilon_{r}$ can drop from 78 to 16 for *E* = 2 × 10^9^ V·m^−1^ [14]. Empirical formulas support this trend [29,30]. However, this points out a conceptual issue: $\varepsilon_{r\;}$ may decrease because of increased ion concentrations, as supported by Hückel’s theory [31], which could mean the field-induced dissociation decreases $\varepsilon_{r\;}$, reversing the assumed causality. Furthermore, the SWE’s exponential sensitivity to *E* results in numerical instability when varying $\varepsilon_{r}$ with *E*: applying Equations (1) and (2) gives 1210 A·m^−2^ at 10^9^ V·m^−1^, but an unrealistic 10^8^ A·m^−2^ at 2 × 10^9^ V·m^−1^. Also, water’s $\varepsilon_{r}$ may drastically decrease in confined environments [32] even without an electric field—yet inserting such values into SWE remains problematic.

### 2.2. Catalytic Protonation–Deprotonation Mechanism

The second mechanism, introduced by Simons [33,34], proposes that WD is catalyzed by membrane functional groups—namely, BH^+^ in the AEM and A^−^ in the CEM—through the following reactions:

B + H_2_O ⇌ BH^+^ + OH^−^, (3)

BH^+^ + H_2_O ⇌ B + H_3_O^+^, (4)

A + H_2_O ⇌ AH + OH^−^, (5)

AH + H_2_O ⇌ A^−^ + H_3_O^+^. (6)

However, this mechanism yields dissociation kinetics that are too slow [28]. This low kinetics prediction led to the development of the Chemical Reaction Model (CRM) [35,36,37], where the electric field is assumed to accelerate protonation reactions between water and fixed charges. Relying on statistical arguments about the field-induced alignment of water molecules and catalytic sites [28,38], the water dissociation enhancement based on the Chemical Reaction Model with its rate constant is given as follows:

(7) $$\;\;\;\;\;\;\;\;\;k_{d}^{cr}\left(E\right)=\;k_{d}^{cr}\left(0\right)\times \;e^{\frac{\alpha FE}{RT}},$$

where $k_{d}^{cr}\left(E\right)\;$ is the field-enhanced WD rate constant due to protonation–deprotonation reactions of water molecules with fixed charges, α is a fitting parameter homogenous to a length, *F* is the Faraday constant (96,485 C·mol^−1^), *E* is the applied field (V·m^−1^), *R* is the universal gas constant (8.314 J·mol^−1^·K^−1^), and $k_{d}^{cr}\left(0\right)$ is the rate constant when no field is applied, given by an Arrhenius expression as follows [28,38]:

(8) $$\;\;\;\;\;\;\;\;\;\;\;\;\;\;\;k_{d}^{cr}\left(0\right)=\;A\times \;e^{-\frac{E_{A}}{RT}}$$

where *A* is the pre-exponential factor, *E_A_* is the activation energy, *R* is the gas constant, and *T* is the temperature. According to CRM, the maximum WD current density *J* in the BPM junction depends on the concentration of catalytic fixed groups $c_{cag}$ and is given by the following equation:

(9) $$J=F\times c_{cag}\times k_{d}^{cr}\left(E\right)\times \delta =F\times c_{cag}\times \;A\times \;e^{-\frac{E_{A}}{RT}}\times e^{\frac{\alpha FE}{RT}}\times \delta ,$$

where all the parameters are defined in the above corresponding relations. The above CRM formulation relies on multiple poorly defined parameters that are difficult to determine independently—the concentration of the catalytic groups, the pre-exponential factor A, and the constant α—thus making it difficult to accurately model ion transport in BPMs. The recent literature [18,38] often identifies the parameter α with the Bjerrum length $l_{b}$, over which a dipole is dissociated into its respective ions in a medium, using an SWE approximation at low fields. However, the SWE exponential formula with $l_{b}$ overestimates the increase in $k_{d}$, leading to unphysical divergences [18].

### 2.3. Alternative Models

Martinez and Farrell [13] used quantum chemical simulations to evaluate $k_{d}$ under strong electric fields. Their results resemble SWE predictions and also rely on a reduction in $\varepsilon_{r}$. Saitta et al. [39] reported water dissociation thresholds of 3.5 × 10^9^ V·m^−1^ and up to 15–20% dissociation at 10^10^ V·m^−1^ using molecular dynamics, but such field strengths far exceed those in BPMs. Hurwitz and Dibiani [25,40] attempted to propose an electrochemical model, where the WD is seen as a redox-like process involving H^+^ and OH^−^ ions by exploiting its heterolytic nature [25,40], thereby justifying Tafel or Butler–Volmer treatments [28]. However, such an analogy lacks a solid basis in BPMs, where no electron transfer occurs, and WD is better described as a chemical reaction, not governed by an electrochemical reaction involving electron transfer at the electronic/electrolyte interface, in the strict sense. Additional proposals invoke semiconductor p–n junction analogies [41,42] or ion solvation kinetics [43], but still fail to quantitatively recover the orders of magnitude required for $k_{d}\left(E\right)$/$k_{d}\left(0\right)$. Recent transport models even omit a specific WD mechanism and assume “sufficiently fast” dissociation [44].

Overall, despite clear evidence that strong fields enhance WD, existing models do not consistently explain this increase. Motivated by the quantitative mismatch summarized above, we propose a power dissipation-based framework in which WD is driven by dissipated field-supplied power to autoprotolysis ions. The resulting picture establishes a quadratic dependence of $\;k_{d}$ on the field E and provides a compact route to device-level predictions.

## 3. Theoretical

### 3.1. Power Dissipation Model for Electric-Field-Driven WD

#### 3.1.1. Framework and Working Hypothesis

In aqueous electrolytes, field-driven transport of ions follows the well-known drift regime [45]. Under the influence of an electric field *E*, an ion of charge *q* experiences an electric force $F_{e}$ proportional to the field *E* as follows:

(10) $$F_{e}\;=\;qE$$

which is opposed by viscous friction $F_{f}$:

(11) $$F_{f}=f\times v$$

where *f* and *v* are the friction coefficient and ion velocity, respectively. Within an extremely short time, these forces balance as follows:

(12) $$\;\;F_{e}\;+\;F_{f}\;=0$$

so the ion moves at a constant drift velocity, *v_d_*, proportional to *E*:

(13) $$v_{d}\;=\;µE,$$

where *µ* is the ionic mobility in (m^2^·V^−1^·s^−1^). Although no kinetic energy accumulates in this steady state, the field continuously injects energy that is entirely dissipated by viscous friction at a rate corresponding to field-supplied power per ion *P* in (J·s^−1^), given by

(14) $$P=F_{e}\times v_{d}=qµE^{2}.$$

Liquid water always contains minority ions, H_3_O^+^ and OH^−^, at very low concentrations, i.e., 10^−7^ mol·L^−1^, at 25 °C due to autoprotolysis:

(15) $$2\;H_{2}O\left(l\right)\;⇌\;H_{3}O_{\left(aq\right)}^{+}\;+\;OH_{\left(aq\right)}^{-}$$

with an equilibrium constant $K_{w}=10^{-14}$ at 25 °C. When an electric field is applied to liquid water, these minority ions are the only species that undergo field-directed motion and dissipate power, whereas neutral H_2_O molecules may merely reorient their dipoles. For BPM-relevant strong fields (E ≳ 10^8^ V·m^−1^), the per-ion dissipated power of H_3_O^+^ and OH^−^ is non-negligible and potentially chemically relevant at the molecular scale. Order-of-magnitude estimates support this plausibility.

At 25 °C, ${µ}_{H3O+}$ = 3.62 × 10^−7^ m^2^·V^−1^·s^−1^ and ${µ}_{OH-}$ = 2.05 × 10^−7^ m^2^·V^−1^·s^−1^ [46]. For a typical BPM field *E* = 10^8^ V·m^−1^, with *q* = 1.6 × 10^−19^ C for both ions H_3_O^+^ and OH^−^, the drift powers, using Equation (14), are defined as follows:

$$P_{H3O+}\;\approx \;5.79\;\times \;10^{-10}\;J\cdot s^{-1}\;\mathrm{and}\;P_{OH-}\approx 3.28\times 10^{-10}\;J\cdot s^{-1}.$$

The molar Gibbs free energy of dissociation $\Delta G_{d}$ (forward reaction in Equation (15)) is obtained from the equilibrium constant $K_{w}$ as follows:

(16) $$\Delta G_{d}=-RT\times ln\left(K_{w}\right)$$

At 25 °C, $\Delta G_{d}$ = 79.9 kJ·mol^−1^, meaning that the theoretical energy required to dissociate one mol of water is 79.9 kJ [47]. The per-molecule dissociation threshold energy $E_{d}$ (in J) required to dissociate one water molecule is obtained from the molar Gibbs free energy of dissociation $\Delta G_{d}$ using the following formula:

(17) $$E_{d}=\frac{\Delta G_{d}}{N_{A}}$$

where *N_A_* = 6.022 × 10^23^ mol^−1^ is Avogadro’s number. At 25 °C, $E_{d}$ ≈ 1.33 × 10^−19^ J. Thus, under *E* = 10^8^ V·m^−1^, each single H_3_O^+^ and OH^−^ ion can, in theory, dissipate enough energy each second to induce.

$\frac{P_{H3O+}\;}{E_{d}}\;$= 4.35 × 10^9^ and $\frac{P_{\mathrm{OH}-}\;}{E_{d}}\;$= 2.46 × 10^9^ potential dissociation events per second, respectively.

In the specific context of BPMs under reverse bias, once the junction is depleted of mobile counter-ions, the residual current is carried by the minority autoprotolysis ions H_3_O^+^ and OH^−^ [5,14]. Because enhanced water dissociation is observed after this minority-carrier regime is established, it is natural to consider WD as a consequence of that pre-existing drift—the real process through which the field supplies power to the system.

Scaling up to a fully hydrated BPM with a surface area *A* = 1 cm^2^ and a junction *δ* = 1 nm, the volume of the junction is *V_j_* = 10^−13^ m^3^, with concentrations of liquid water in the junction and the minority autoprotolysis ions of $c_{H2O,j}$ = 55.5 mol·L^−1^ and *cᵢₒₙₛ* = 10^−7^ mol·L^−1^, respectively. The total power dissipated by all the H_3_O^+^ and OH^−^ ions present in the junction’s volume *V_j_* can be expressed as follows:

(18) $$P_{total}=n_{i}q\left({µ}_{H3O^{+}}\;+\;{µ}_{{\mathrm{OH}}^{-}}\right)E^{2}\times V_{j},$$

where $\;n_{i}$ in (m^−3^) is the volumetric density of ions H_3_O^+^ and OH^−^, given by the following:

(19) $$n_{i}=c_{i}\times N_{A},$$

where $c_{i}$ is the concentration of ions. $n_{i}$ is equal (i.e., $n_{H3O+}=n_{OH-}=\;6.022\times 10^{19}\;m^{-3})$, since they have the same concentration. The volume *V_j_* contains 3.3 × 10^15^ water molecules and 6.022 × 10^6^ H_3_O^+^ and OH^−^ ions each. The total dissipated power for *E* = 10^8^ V·m^−1^ is $P_{total}$ ≈ 5.46 × 10^−3^ J·s^−1^. The corresponding number of potential dissociation events per second is $\frac{P\mathrm{total}\;}{E_{d}}=\;$4.1 × 10^16^, meaning that the entire water content of the junction could, in theory, dissociate in less than one second. Furthermore, given the thermal conductivity of water (κ ≈ 6 × 10^−3^ J·s^−1^·cm^−1^·K^−1^) and the nanometric thickness of the junction (*δ* = 1 nm), a simple steady-state heat-flux estimate is given using the following expression [48]:

(20) $$P_{\mathrm{total}}=\frac{\kappa \times A\times \Delta T}{\delta /2},$$

which gives a temperature rise Δ*T* ≈ 5 × 10^−8^ K, showing that Joule heating is negligible at the junction scale and the system can be treated as effectively isothermal under the conditions considered. In this isothermal limit, the power dissipated by the drift of autoprotolysis ions corresponds, in the sense of non-equilibrium thermodynamics, to the internal entropy production $d_{i}S$, according to the following equation [49]:

(21) $$P_{\mathrm{diss}}=T\;\left(d_{i}S/dt\right).$$

Thus, the dissipated power in the nanometric junction is interpreted as local entropy production, which can drive microscopic molecular rearrangements and provides a physically consistent basis for relating dissipation to field-assisted water dissociation.

Building on the estimates above, our working hypothesis is that the dissipated power of the drifting autoprotolysis ions (H_3_O^+^, OH^−^) under strong electric fields is more than sufficient to drive new water dissociation events per second. Thus, the sudden increase in the WD rate observed in BPMs at high electric fields is an indirect ion-mediated consequence of power dissipation rather than a direct field action on neutral water molecules. Therefore, in the BPM junction, the intense electric field increases the effective dissociation probability by amplifying the activation frequency attempts powered by drift dissipation above the dissociation energy *E_d_*. Accordingly, we postulate that the WD rate constant $k_{d}\left(E\right)$ is proportional to the ratio of the total dissipated power $P_{total}$ over $E_{d}$: $k_{d}\left(E\right)$ ∝ $\;\frac{P_{total}\;}{E_{d}}$.

#### 3.1.2. Analytical Formulation of *k_d_*(*E*)

We interpret the WD rate constant $k_{d}$ as a frequency of energy delivery relative to the activation barrier. Let the power density *w* (J·s^−1^·m^−3^), deposited by all field-driven ions H_3_O^+^ and OH^−^, be

(22) $$w=n_{H3O+}\times P_{H3O+}+\;n_{OH-}\times P_{OH-},$$

where $\;n_{H3O+}\;$ and $\;n_{OH-}$ are the volumetric densities of H_3_O^+^ and OH^−^, respectively; $P_{H3O+\;}$ and $P_{OH-\;}$ are the dissipated powers of H_3_O^+^ and OH^−^, respectively. Since the concentrations of H_3_O^+^ and OH^−^ in pure water are equal (i.e., $c_{\mathrm{ions}}=c_{H3O+}=c_{\mathrm{OH}-}$ = 10^−7^ mol·L^−1^), their volumetric density is also equal ($n_{\mathrm{ions}}=n_{H3O+}=n_{\mathrm{OH}-}$). Thus,

(23) $$w=n_{\mathrm{ions}}\times (P_{H3O+}+P_{OH-}).\;$$

Dividing by the threshold dissociation energy per event $E_{d}$ gives a volumetric event rate $R_{vol}\;$ (s^−1^·m^−3^) as follows:

(24) $$R_{vol}=\frac{w}{E_{d}}.$$

The per-molecule (pseudo-first-order) rate constant $k_{d}\left(E\right)$ in (s^−1^) is then obtained by normalizing with the number density of water molecules $\;n_{H2O,l}$ as follows:

(25) $$k_{d}\left(E\right)=\frac{R_{vol}}{\;n_{H2O,l}}=\left(\frac{n_{ions}}{n_{H2O,l}}\right)\times \frac{\left(P_{H3O+\;}+P_{OH-\;}\right)}{E_{d}}.$$

Since

(26) $$\left(\frac{n_{ions}}{n_{H2O,l}}\right)=\left(\frac{c_{ions}\times N_{A}}{c_{H2O,l}\times N_{A}}\right)=\left(\frac{c_{ions}}{c_{H2O,l}}\right)$$

and replacing $P_{H3O+\;}$ and $P_{OH-\;}$ by their respective formulas, i.e.,

(27) $$P_{H3O+}=q{µ}_{H3O^{+}}E^{2},$$

and

(28) $$P_{OH-}=q{µ}_{{\mathrm{OH}}^{-}}E^{2}$$

we obtain the final expression of $k_{d}$(*E*) as follows:

(29) $$k_{d}\left(E\right)=\left(\frac{c_{ions}}{c_{H2O,l}}\right)\times \frac{q\;\times \;\left({µ}_{H3O^{+}}\;+\;{µ}_{{\mathrm{OH}}^{-}}\right)}{E_{d}}\;\times \;E^{2},$$

where

$k_{d}\left(E\right)$: Dissociation rate constant of water molecules as a function of the field *E* in (s^−1^);
*cᵢₒₙₛ:* Concentration of H_3_O^+^ and OH^−^ ions in water (10^−7^ mol·L^−1^ at 25 °C);
$c_{H2O,l}$: Concentration of free liquid water (55.5 mol·L^−1^ at 25 °C);
*q*: Absolute electric charge of H_3_O^+^ and OH^−^ ions (*q* = 1.6 × 10^−19^ C);
$\mu_{H3O+}$: Ionic mobility of the hydronium ion H_3_O^+^ (3.62 × 10^−7^ m^2^·V^−1^·s^−1^ at 25 °C);
$\mu_{OH-}$: Ionic mobility of the hydroxide ion OH^−^ (2.05 × 10^−7^ m^2^·V^−1^·s^−1^ at 25 °C);
  *E*: The electric field in the BPM junction in (V·m^−1^);
$E_{d}$: Threshold dissociation energy per water molecule in (J), ($E_{d}$ = 1.33 × 10^−19^ J at 25 °C).

This formulation reveals $k_{d}$ is a simple quadratic dependence on electric field *E*, representing a significant departure from previously proposed exponential models. It provides a clear analytical link between physicochemical parameters—elementary charge, ion mobility, dissociation energy, and molar concentrations—and the dissociation rate, without relying on empirical constants or catalytic assumptions. This analytical form enables a direct estimation of the dissociation rate $k_{d}$ as a function of the electric field *E* applied at the BPM junction.

We note that the mobilities of H_3_O^+^ and OH^−^ in water are obtained from [50]:

(30) $$µ\;=\Lambda_{0}/F,$$

where $\Lambda_{0},$ is the limiting molar conductivity at infinite dilution in (S·m^2^·mol^−1^) and *F* is the Faraday constant (96,485 C·mol^−1^). It was shown that $\Lambda_{0}$ is independent of the field strength [51], as are the mobilities. In addition, studies reported a negligible influence of high electric fields on the viscosity *η* of solvents [52,53]; as per Stokes–Einstein relation *µ* ∝ 1/*η* [46], the effect is also negligible on µ.

Furthermore, Equation (29) carries an implicit temperature dependence (*T*) via three parameters: *µ*, which increases with *T* via *η* [54], and *cᵢₒₙₛ* and $E_{d}$, which also increase with *T* via the Nernst relation (Equation (16)) [55]. Overall, the model relies solely on measurable, field-independent quantities at a constant temperature. By substituting standard values at 25 °C into Equation (29), we obtain the following simplified expression for the water dissociation rate:

(31) $$k_{d}\left(E\right)\;=1.23\times 10^{-15}\cdot E^{2}.$$

This equation will be used for comparison benchmarks with SWE at 25 °C in Section 3.2.1.

#### 3.1.3. Junction J-V Relation Derived from $k_{d}\left(E\right)$

Under reverse bias, the current in a BPM primarily results from ion migration generated by water dissociation at the junction, which is captured by Equation (1). Let *U_j_* denote the junction voltage drop across the active region of thickness *δ*. Assuming a quasi-uniform field in the junction yields the following:

(32) $$E=\frac{U_{j}}{\delta }$$

By replacing *E* of Equation (29) with that of Equation (32) and inserting Equation (29) for $k_{d}$(*E*) into Equation (1), we obtain

(33) $$J=\frac{F\times f\times q\times c_{ions}\times \left({µ}_{H3O^{+}}+{µ}_{{\mathrm{OH}}^{-}}\right)\;}{\delta \times E_{d}}\times U_{j}^{2},$$

where

(34) $$f=\frac{c_{H2O,j}}{c_{H2O,l}}$$

is the ratio of the effective water concentration in the junction $c_{H2O,j}\;$ to the concentration $c_{H2O,l}$ of free liquid water. The parameter *f* is a dimensionless factor representing the water volume fraction in the BPM junction [56,57,58], which varies from 0 (no water) to 1 (fully hydrated junction). Let

(35) $$K=\frac{F\times f\times q\times c_{ions}\times \left({µ}_{H3O^{+}}+{µ}_{{\mathrm{OH}}^{-}}\right)}{\delta \times E_{d}};$$

then, Equation (33) simplifies to

(36) $$J=K\times U_{j}^{2},$$

where *K* is in (mA·cm^−2^·V^−2^), if *J* and *U_j_* are in (mA·cm^−2^) and volts (V), respectively.

Assuming a homogeneous BPM with a nanometric junction *δ* = 1 nm, fully hydrated (*f* = 1), and standard physical constants at 25 °C, Equation (36) gives

(37) $$J\approx \;660\;\times \;U_{j}^{2}$$

We emphasize that Equation (36) is a junction-level law; in experiments, what is controlled is the external cell voltage $E_{cell}$. The experimental determination of *U_j_* from $E_{cell}$ is described in the experimental section.

### 3.2. Theoretical Results

#### 3.2.1. *k_d_*(*E*) Enhancement and Comparison with SWE

**Figure 1:**
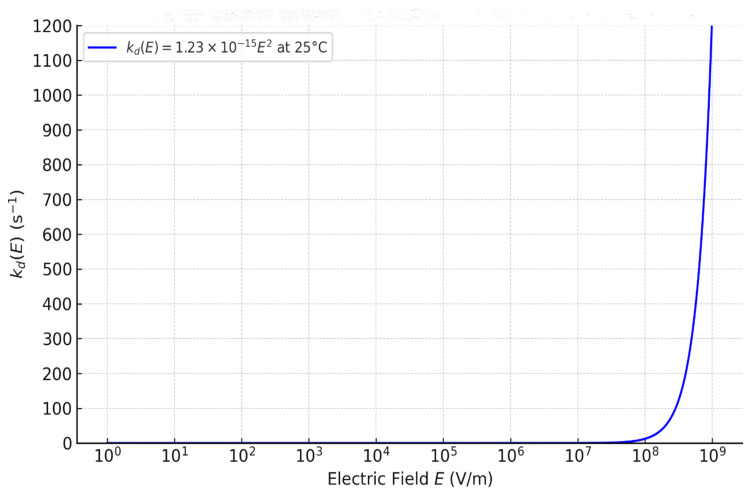
Curve of the evolution of $k_{d}\left(E\right)$ according to $k_{d}$(*E*) = $1.23\times 10^{-15}\cdot E^{2}$.

Figure 1 shows the resulting curve $k_{d}$(*E*) at 25 °C from Equation (31), at a 0–100 s^−1^ y-axis scale. Note that this scale compresses the low-field region: although Equation (31) yields a strictly quadratic onset from *E* = 0, $k_{d}$(*E*) remains <1 s^−1^ up to *E* ≈ 2.8 × 10^7^ V·m^−1^, so the curve appears nearly flat at this scale (a zoom reveals the monotonic rise).

The profile closely resembles typical BPM *J–V* behavior, where an initial low-conductivity region is followed by a sharp increase beyond a threshold. The crossover where $k_{d}\left(E\right)$ = $k_{d}\left(0\right)$ occurs at E* ≈ 1.43 × 10^5^ V·m^−1^. For a nanometric BPM junction (*δ* = 1 nm), this field corresponds to a voltage of 0.14 mV. At fields below E*, the overall dissociation is governed by the thermal constant $k_{d}\left(0\right)$. From E* up to ∼2.8 × 10^7^ V·m^−1^ (*U_j_* ≈ 28 mV for *δ* = 1 nm), $k_{d}$(*E*) exceeds $k_{d}\left(0\right)$ but remains <1 s^−1^; rates are still weak, indicating a non-activated regime where the field is insufficient to trigger significant dissociation. A sharp transition emerges above 10^8^ V·m^−1^, corresponding to voltages *U_j_* ≳ 0.1 V in the nanometric junction, marking the onset of an activated regime where the field supplies enough power to accelerate dissociation. To quantify this transition, values of $k_{d}$(*E*) and the amplification ratio $k_{d}\left(E\right)/k_{d}\left(0\right)\;$ across the activated field range are reported in Table 1.

These values show consistent increases in $k_{d}\left(E\right)/k_{d}\left(0\right)$, from ~5 × 10^5^ to ~2 × 10^8^, over the field range 10^8^–2 × 10^9^ V·m^−1^, aligning with experimental requirements for sustaining current densities of 10 to several hundred mA/cm^2^. The quadratic form thus captures the field activation effect realistically: the progression is rapid yet stable, avoiding numerical divergence and remaining compatible with electrochemical integration through Equation (1).

To further contextualize our model, we compare it with the SWE model. Table 2 presents the $k_{d}\left(E\right)/k_{d}\left(0\right)$ ratios for each model at two representative fields (10^8^ and 2 × 10^9^ V·m^−1^), corresponding to 0.1 V and 2 V across a 1 nm thick BPM junction. For the SWE model, both the constant permittivity ($\varepsilon_{r}$ = 78) and field-dependent permittivity cases are considered ($\varepsilon_{r}=$ 78 to 16 for *E* = 10^8^ to 2 × 10^9^ V·m^−1^). Two more scenarios with fixed $\varepsilon_{r}$ (10 and 5) are considered to account for possible nanoconfinement effects.

The results highlight the fundamental disparities between the models. In the SWE model, small field variations cause drastic shifts in $k_{d}$. At *E* = 10^8^ V·m^−1^, SWE remains weak across both $\varepsilon_{r}\;$ choices, whereas the quadratic law already yields ~5 × 10^5^. The SWE model with constant $\varepsilon_{r}$ shows a limited increase (<5 × 10^4^) at 2 × 10^9^ V·m^−1^, which is insufficient for supporting BPM current densities. Its variable-$\varepsilon_{r}$ version improves predictions but introduces instability, with a sharp divergence at 2 × 10^9^ V·m^−1^. Nanoconfinement effects do not alter the qualitative conclusion that SWE either underestimates activation at 10^8^ V·m^−1^ or becomes unstable at higher fields. By contrast, our quadratic model exhibits a large yet controlled amplification, without runaway divergence over the same field window *E* ∈ [10^8^–2 × 10^9^] V·m^−1^, providing a robust basis for BPM-relevant modeling.

#### 3.2.2. Theoretical J-V Curve of a Nanometric Fully Hydrated BPM Junction

**Figure 2:**
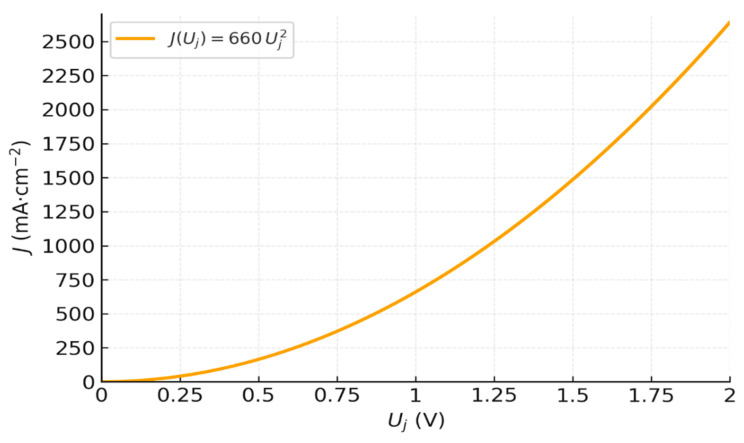
Theoretical *J–U_j_* curve of a nanometric fully hydrated BPM junction, obtained from *J* = 660 $U_{j}$^2^.

Figure 2 shows the resulting *J–U_j_* curve of a fully hydrated BPM nanometric junction (*δ* = 1 nm) (*f* = 1) at 25 °C from Equation (37).

The curve features a smooth, continuous increase in current, with no apparent threshold. At low voltages, the current remains low but non-zero, reflecting a gradual activation of dissociation. As the voltage increases, the field intensifies, and the current increases quadratically. This curve defines a theoretical upper bound (homogeneous, fully hydrated 1 nm junction; uniform field; no interfacial resistance). It thus serves as a universal benchmark for interpreting experiments and diagnosing losses (partial hydration *f* < 1, thicker effective junction *δ* > 1 nm, ohmic drops, and electrode overpotentials), which are analyzed in Section 4 and discussed later. It should be noted that *U_j_* = 1 V already gives a theoretical current of *J* = 660 mA·cm^−2^. At *U_j_* = 1.75 V, we approach 2 A·cm^−2^, which corresponds to the typical current of PEM electrolyzers. It can also be noted that the current density of *J* = 100 mA·cm^−2^ corresponds to a voltage of *U_j_* = 0.38 V, i.e., a field *E* = 3.8 × 10^8^ V·m^−1^, for a 1 nm BPM junction. The corresponding $k_{d}\left(E\right)/k_{d}\left(0\right)$ ratio obtained by our model is 7 × 10^6^, a value fully consistent with BPM observations from Section 1. This agreement further supports the predictive strength and physical consistency of our quadratic model.

## 4. Materials and Experimental Methods

### 4.1. Membrane and Electrodes

A commercial bipolar membrane (Fumasep^®^ FBM, acquired from FuelCell Store, Bryan, TX, USA) was cut into samples of 1.0 cm^2^ active area. The acidic- and alkaline-side working electrodes were Pt foil (1.0 cm^2^) and Ni foam (1.0 cm^2^), respectively, both obtained from Sigma Aldrich (Oakville, ON, Canada). They were cleaned using acetone, deionized water, and alcohol before use. These electrodes are commonly used for BPM water electrolysis characterization [11,59].

### 4.2. Electrolytes, Cell Type, and Components

Experiments were performed in an H-cell (Stony Lab, Nesconset, NY, USA) with two liquid compartments separated by the BPM. The acidic and alkaline compartments contained 0.5 M of H_2_SO_4_ (pH = 0) and 1.0 M of NaOH (pH = 14), respectively. The inter-electrode spacing was 10 cm (non-zero-gap); the electrode–membrane distance was 5 cm on each side. Unless stated otherwise, measurements were conducted at 25 °C. We emphasize that this is an electrolysis configuration (acid|BPM|alkali), not an electrodialysis stack.

### 4.3. Electrochemical Instrumentation and Measurement Protocols

Linear sweep voltammetry (LSV) was performed in a two-electrode configuration on the full BPM cell, using a Solartron 1287A potentiostat/galvanostat (Scribner Associates Inc., Southern Pines, NC, USA). The cell voltage $E_{cell}$ was swept at 5 mV·s^−1^. Electrochemical impedance spectroscopy (EIS) was performed with a VersaSTAT 4A (Ametek, Oak Ridge, TN, USA), using a small-signal sinusoidal perturbation (10 mV_rms_) over a log-spaced frequency sweep from 1 MHz down to 0.1 Hz. For the two-compartment BPM cell, the global series resistance $R_{s}$ was obtained from the high-frequency real-axis intercept of the Nyquist plot; values are reported in Ω·cm^2^ after normalization by 1.0 cm^2^ geometric area. Data and extraction details are provided in Section 5. LSVs were collected as raw curves and, when specified, as $iR_{s}$-corrected curves using the $R_{s}$ obtained from EIS.

### 4.4. Three-Electrode Configuration Characterization of the Hydrogen Evolution Reaction (HER) and of the Oxygen Evolution Reaction (OER)

To quantify electrode kinetics independently of the membrane cell, we performed three-electrode configuration measurements for the Hydrogen Evolution Reaction (HER) and the Oxygen Evolution Reaction (OER) in their respective electrolytes. In this study, the hydrogen gas pressure is 1 atmosphere (P_H2_ = P_O2_= 1 atm). The following half-reactions are considered in their respective compartment with their equilibrium potentials referenced to the Reversible Hydrogen Electrode (*RHE*) whose potentials at standard conditions are $E_{HER}^{o}$ = 0 V vs. *RHE* for the HER at pH = 0, and $E_{OER}^{o}$ = 1.23 V vs. *RHE* for the OER at pH = 14, respectively [47]:

In the acidic compartment, the following Hydrogen Evolution Reaction (HER) occurs:

(38) $$4H^{+}+4e^{-}\to 2H_{2}$$

The dependence of the equilibrium potential of the reaction on the pH is

(39) $$E_{HER}^{eq}=E_{HER}^{o}-0.059\times pH=-0.059pH$$

pH = 0, P_H2_ = 1, and T = 298 K are standard conditions.

(40) $$E_{HER}^{eq}=0.00\;\mathrm{V vs}.\mathrm{SHE}$$

In the alkaline compartment, the following Oxygen Evolution Reaction (OER) occurs:

(41) $$4OH^{-}\to O_{2}+2H_{2}O+4e^{-}$$

The dependence of the equilibrium potential of the reaction with the pH is

(42) $$E_{HER}^{eq}=E_{OER}^{o}-0.059\times pH=1.23-0.059pH$$

In this compartment, pH = 14.

(43) $$E_{OER}^{eq}=0.401\;\mathrm{V vs}.\mathrm{SHE}$$

For the HER in 0.5 M H_2_SO_4_, the reference electrode was Hg/HgSO_4_; for the OER in 1.0 M NaOH, the reference electrode was Hg/HgO. All potentials were converted to *RHE* using fixed offsets (Hg/HgSO_4_ (sat.) = 0.64 V vs. *RHE*; Hg/HgO = 0.098 V vs. *RHE*). The Solartron 1287A applied 100% *iR* compensation in these three-electrode tests (note: this *R* is not the same as the two-compartment BPM cell $R_{s}$). Overpotentials were computed as follows:

(44) $$\eta_{HER}=E_{HER}-E_{HER}^{eq}$$

(45) $$\eta_{OER}=E_{OER}-E_{OER}^{eq},$$

where $E_{HER}$ and $\;E_{OER}\;$ are the applied potential to the electrode of the Hydrogen Evolution Reaction in the acidic compartment and of the Oxygen Evolution Reaction in the alkaline compartment during the tests, respectively. Each of the potential vs. current plots displays a Tafel behavior in a certain range of potential according to the following:

(46) $$\eta_{HER}=\;b_{1}\log\left(\frac{J_{1}}{J_{0,HER}}\right)$$

for the HER and

(47) $$\eta_{OER}=b_{2}\log\left(\frac{J_{2}}{J_{0,OER}}\right)$$

for the OER.

The respective HER and OER Tafel slopes $b_{1}$ and $b_{2}$ and their corresponding exchange current densities $J_{0,HER}$ and $J_{0,OER}\;$ were obtained via linear fits of the above Tafel forms applied in the appropriate overpotential ranges where *E-log J* is quasi-linear. In particular, we used the following:

− HER: Low overvoltage window $\eta_{HER}\;$ ∈ [−65, –45] mV (cathodic currents, after taking into account the value of the reference electrodes and *iR*-compensation);
− OER: Window $\eta_{OER}\;$ ∈ [+0.20, +0.35] V (controlled by activation regime, *iR*-compensated).

The numerical values of the extracted Tafel slopes and exchange current densities are reported in Section 5, together with the corresponding polarization and Tafel plots (including the fitted windows).

### 4.5. BPM’s Junction Voltage Accounting

For comparison to the theoretical junction law, we computed the junction drop *U_j_* from the external cell bias $E_{cell}$ by removing equilibrium, series resistance, and overpotentials contributions [57,58]:

(48) $$U_{j}≃E_{cell}-E_{cell}^{rev}-iR_{s}-\left|\;\eta_{HER}\right|-\eta_{OER},$$

where $\;E_{cell}^{rev}$ is the reversible equilibrium voltage of the cell:

(49) $$E_{cell}^{rev}=E_{OER}^{o}-\;E_{HER}^{o}$$

Since $E_{HER}^{0}=0.00\;$V vs. *RHE* and $E_{OER}^{0}=1.23\;$V vs. *RHE*, then $E_{cell}^{rev}=1.23$ V vs. *RHE*. The LSV-derived current densities were then compared to the model prediction Equation (37). All fits and goodness-of-fit metrics are presented in Section 5.

## 5. Results and Discussion

**Figure 3:**
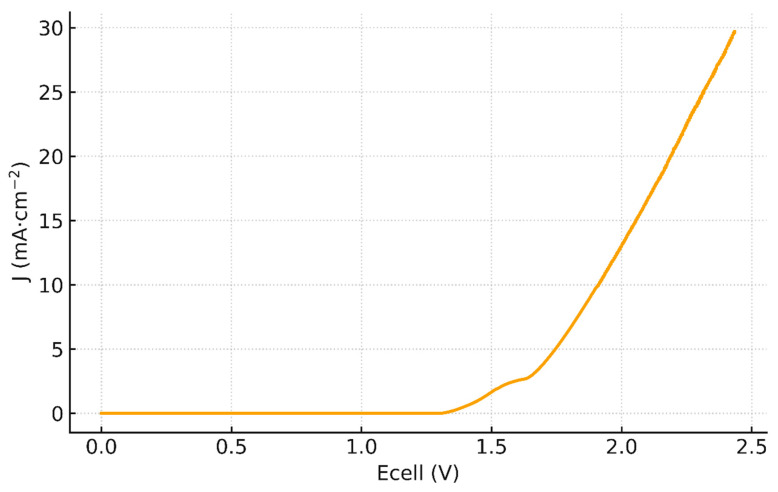
Raw *J* vs. $E_{cell}$ curve of Fumasep^®^ FBM obtained from LSV measurement.

Figure 3 shows the raw *J* vs. $E_{cell}$ curve of Fumasep^®^ FBM obtained from LSV (two-compartment FBM cell, ΔpH = 14). A measurable current appears near 1.36 V (sub- mA·cm^−2^), while the 1 mA·cm^−2^ onset occurs at ≈ 1.42 V. The curve exhibits three salient regions: first, a linear ohmic-like rise between 1.36 and 1.54 V, followed by a quasi-stationary plateau between 1.55 and 1.62 with an average limiting current density *J_lim_* ≈ 2.19 mA·cm^−2^, and then a transition into an over-limiting regime beyond ∼1.62 V, where the current leaves the shelf and increases rapidly. This morphology is the canonical BPM signature at low current densities [3,44]: an initial rise, a limiting shelf associated with internal depletion in the ion-exchange layers, then take-off as the junction field grows and water dissociation (WD) becomes dominant.

**Figure 4:**
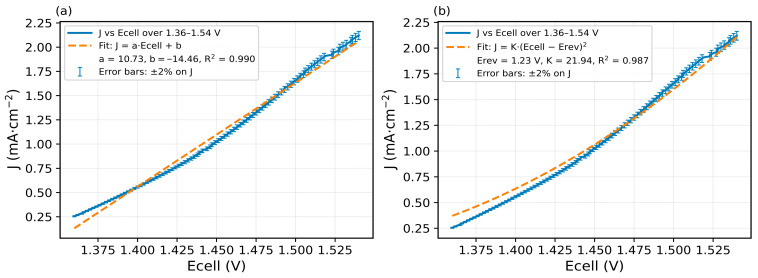
Fits of *J* vs. *Ecell* over 1.36–1.54 V: (**a**) linear fit $J=K.E_{cell}+a$; (**b**) quadratic fit $J\;\mathrm{vs}.\;{\left(E_{cell}-E_{cell}^{rev}\right)}^{2}$.

**Figure 5:**
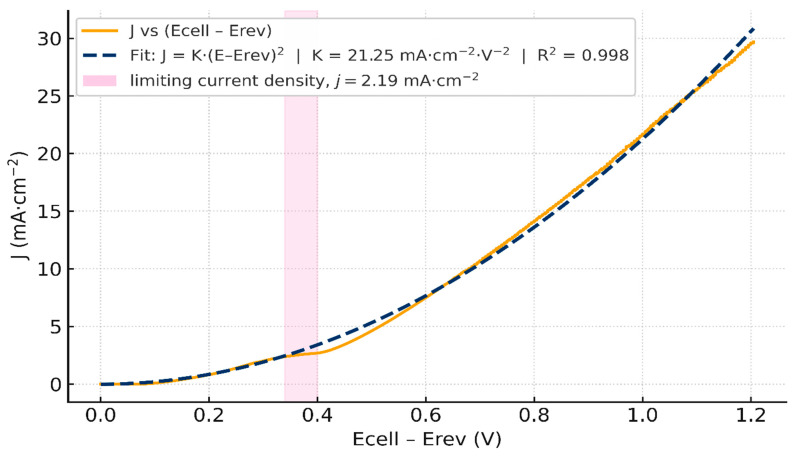
Variation in the current density *J* vs.$\;E_{cell}-E_{cell}^{rev}$, quadratic fit *J* vs. ${\left(E_{cell}-E_{cell}^{rev}\right)}^{2}$ and limiting-current density *J* = 2.19 mA·cm^−2^.

Importantly, a linear fit over 1.36–1.54 V (Figure 4a) is excellent with slope 10.73 mA·cm^−2^·V^−1^ and $R^{2}=0.990$, indicating an ohmic behavior governed by co-ion transport. Yet when we re-plot the same data against $E_{cell}-E_{rev}$ (Figure 5), a quadratic form fits equally well on that narrow span. This apparent paradox suggests that the low-bias current is composite: a nascent WD channel already contributes below 1 mA cm^−2^, as emphasized by recent literature [44], while a residual co-ion transport path (imperfect permselectivity through the ion-exchange layers) adds an ohmic-like component. By contrast, the quasi-plateau 1.55–1.62 V (with *J_lim_* ≈ 2.19 mA·cm^−2^) is not captured by a pure quadratic law (Figure 5) and marks the limiting-current shelf typical of BPMs at low current densities. For completeness, a quadratic fit *J* vs. $\;{\left(E_{cell}-E_{cell}^{rev}\right)}^{2}$ restricted to 1.36–1.54 V with (K = 21.94 mA·cm^−2^·V^−2^, $R^{2}=0.987$) is provided in (Figure 4b). Error bars (±2% on J) are included in Figure 4 to indicate the typical experimental uncertainty in current density in this narrow voltage window.

**Figure 6:**
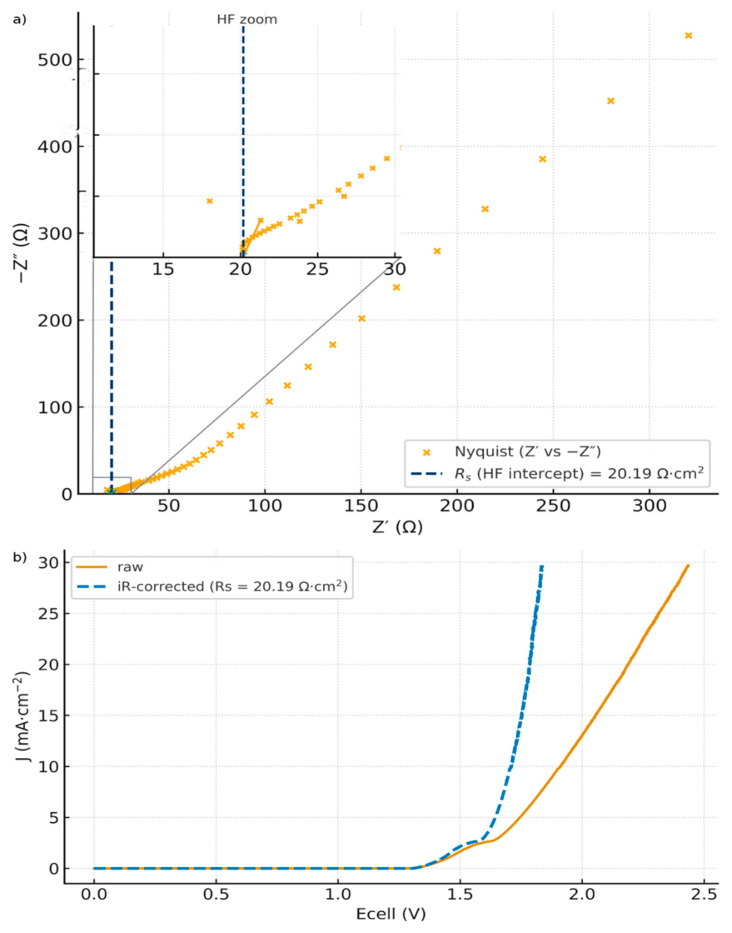
(**a**) Nyquist plot from (EIS) of the FBM H-cell (VersaSTAT 4A) with Zoom on HF-intercept $R_{s}$ = 20.19 Ω·cm^2^; (**b**) raw *J* vs. *Ecell* curves (LSV) for the FBM H-cell together with *iRs*-corrected by the cell-level $R_{s}$ = 20.19 Ω·cm^2^.

Subtracting only the equilibrium reversible voltage ($U_{j}$ ≃ $E_{cell}-\;E_{cell}^{rev}$) already yields an excellent quadratic fit, $R^{2}=0.998$ with a prefactor *K* ≃ 21.25 mA·cm^−2^·V^−2^, as shown in Figure 5. This supports our quadratic model and means that the cell current is mainly dominated by WD. The low prefactor compared to the theoretical *K* = 660 reflects the fact that bulk ohmic loss and electrode overpotentials still remain in the trace. This supports the importance of making the ohmic loss and overvoltage correction, i.e., Equation (48). Figure 6a shows the Nyquist plot obtained from the electrochemical impedance spectroscopy (EIS) measurement. The area-normalized series resistance is extracted from the high-frequency real-axis intercept: $R_{s}$ = 20.19 Ω·cm^2^. Figure 6b shows the corresponding *iRs*-corrected *J* vs. *E_cell_* curve.

**Figure 7:**
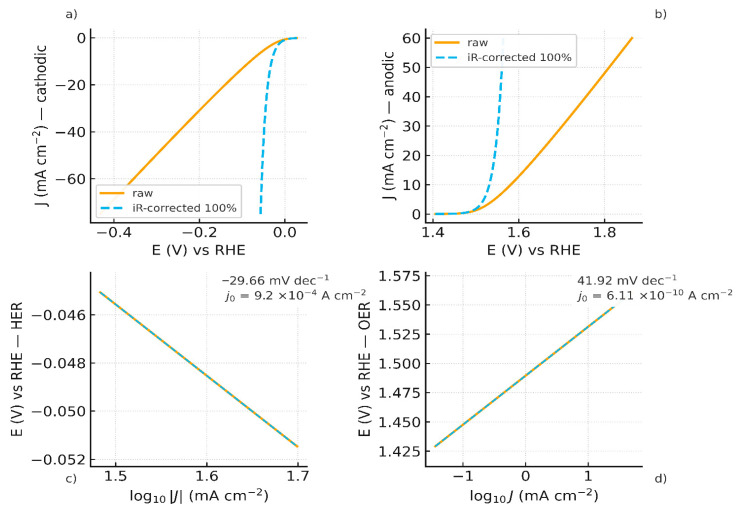
(**a**) HER polarization curves (raw and *iR*-corrected): Pt in 0.5 M H_2_SO_4_ vs. Hg/HgSO_4_ (reported vs. RHE); (**b**) OER polarization curves (raw and *iR*-corrected): Ni-foam in 1.0 M NaOH vs. Hg/HgO (vs RHE); (**c**) HER Tafel plot $b_{1}$ = −29.66 mV·dec^−1^; $J_{0,HER}$ = 9.20 × 10^−4^ A·cm^−2^; (**d**) OER Tafel plot $b_{2}$ = 41.92 mV·dec^−1^; $J_{0,OER}$ = 6.11 × 10^−10^ A·cm^−2^.

Independent three-electrode HER and OER polarization curves, each with its own iR-correction (not the FBM-cell $R_{s}$), are shown in Figure 7a,b, respectively. These datasets supply $\;\eta_{HER}$ and $\eta_{OER}$ used in the junction mapping.

**Figure 8:**
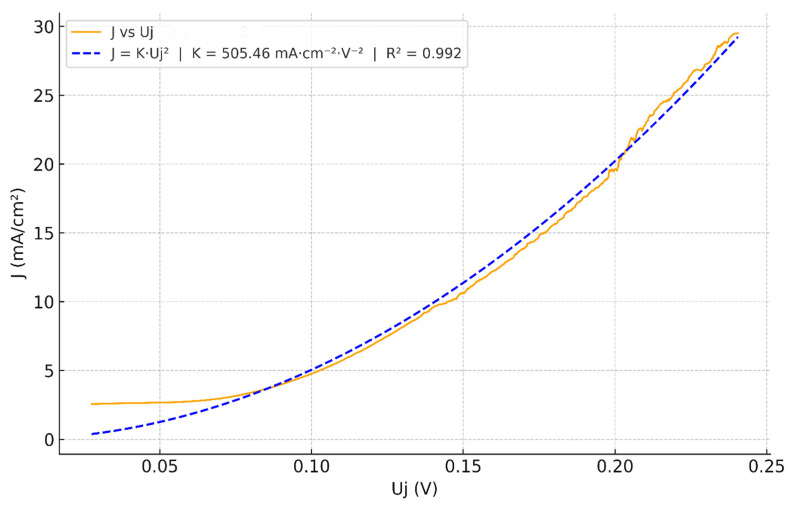
Quadratic fit of J vs. Uj curve obtained from Equation (33): $U_{j}$ ≃ $E_{cell}-E_{cell}^{rev}-iR_{s}-\left|\;\eta_{HER}\right|-\eta_{OER}$.

Independent three-electrode characterizations provided a Tafel slope $b_{1}$ of −29.66 mV·dec^−1^ and an exchange current density $J_{0,HER}$ of 9.2 × 10^−4^ A·cm^−2^ for HER on Pt (Figure 7c). While for OER on Ni foam, a Tafel slope $b_{2}\;$ of 41.92 mV·dec^−1^ and an exchange current density $J_{0,OER}$ of 6.11 × 10^−10^ A·cm^−2^ were obtained (Figure 7d). These values are consistent with literature values for the respective HER and OER on these materials [60,61]. These values are used for the overpotential corrections in the full junction mapping, whose results are shown in Figure 8.

After full junction mapping using Equation (48), the obtained *J* vs. *U_j_* curve of the junction voltage collapses onto a robust quadratic law, $R^{2}=0.992$ with a much larger prefactor *K* ≃ 505.46 mA·cm^−2^·V^−2^, as shown in Figure 8. We notice that in a narrow window *U_j_* ∈ [0, 0.07] V, the current remains nearly constant at *J* ≈ 2.2 mA·cm^−2^, in excellent agreement with the limiting current measured on the raw LSV *J_lim_* ≈ 2.19 mA·cm^−2^. This flat shoulder reflects the transport-limited baseline that precedes strong field-assisted water dissociation (WD) in BPMs; it does not contradict the junction quadratic law. Subtracting this constant baseline would force the curve through the origin and numerically reduce the zero-intercept prefactor *K* (a generic effect of vertical offsets in least-squares), but adds no physical information; therefore, we only report the full-mapping result in Figure 8 and interpret the small offset as the limiting baseline with minor reconstruction offsets expected at a very low junction field.

The prefactor for Fumasep^®^ FBM, *K* ≃ 505.46 mA·cm^−2^·V^−2^, is ~76.6% of the ideal *K* = 660 mA·cm^−2^·V^−2^ estimated for a 1 nm, fully hydrated junction (Section 3.1.3). Since the Fumasep^®^ FBM is homogeneous [62], a *δ* ≈ 1 nm active region is plausible (Section 1). Hence, the observed prefactor gap can be reasonably attributed to the water volume fraction *f*, as reflected in Equation (35). Beyond hydration, the gap can also be explained by a slight deviation in the effective junction thickness from the 1 nm benchmark (e.g., δ≳1.1−1.3 nm, or conversely <1 nm) or a dissipation efficiency α capturing non-productive channels, without affecting the quadratic law. Overall, the FBM junction exhibits a high-quality quadratic dependence of *J* on *U_j_* ($R^{2}\;$≈ 0.99), with a high prefactor *K* after full junction mapping. These results provide a proof-of-principle of the quadratic $k_{d}$(*E*) dependence anticipated by our power dissipation model.

The above experimental results clearly indicate that our proposed model provides a novel power dissipation framework for interpreting WD enhancement under strong electric fields in BPMs with nanometric junctions. The central premise is that the dissipated power of drifting autoprotolysis ions H_3_O^+^ and OH^−^ under a field increases the propensity for heterolytic O–H bond cleavage. Thus, these rare ions act as indirect agents promoting the dissociation of new water molecules. The originality of our approach lies in expressing $k_{d}$(*E*) as proportional to the power dissipated by migrating H_3_O^+^ and OH^−^ ions, thus directly linking field intensity to molecular bond breaking via power dissipation. A direct consequence is a quadratic dependence of the WD rate on field strength $k_{d}$(*E*) ∝ $\;E^{2}$, which we formalized with Equation (29).

Built on fundamental physical principles, our analytical formulation is simple and requires no adjustable parameters; furthermore, it avoids the fragile/explosive behavior of exponential laws at 10^8^–2 × 10^9^ V·m^−1^, yet still delivers the orders of magnitude required for BPM operation. In our comparison (Table 2), SWE is either too weak (constant $\varepsilon_{r}$) or numerically unstable (field-dependent $\varepsilon_{r}$), whereas the quadratic law gives a large but bounded amplification $k_{d}\left(E\right)$/$k_{d}\left(0\right)$~5 × 10^5^ at 10^8^ V·m^−1^ and ~2 × 10^8^ at 2 × 10^9^ V·m^−1^. To our knowledge, this is the first analytical model capable of quantitatively predicting the experimental increase in $k_{d}\left(E\right)$/$k_{d}\left(0\right)$, as observed in the literature. This alone marks a major advancement compared to existing models such as SWE and CRM, which often rely on semi-empirical formulations or are unable to predict the correct order of magnitude of the dissociation rate enhancement.

While Onsager’s formulation of SWE may yield a near-quadratic trend at low fields, this regime is outside the range relevant to BPMs with nanometric junctions, where electric fields exceed 10^8^ V·m^−1^. Moreover, in the SWE, this scaling emerges from a series expansion and is not the central feature of the model; in contrast, in our formulation, it is intrinsic, directly derived from power dissipation, and remains valid over a broader range of practical fields in BPMs. Interestingly, a quadratic field dependence was briefly considered by Simons and Khanarian in their 1978 study [48], where they experimentally reported a quadratic *J*–*V* response across a neutral junction 60 nm to 1 µm thick. However, they dismissed this scenario not on theoretical grounds, but because the junction thickness in their setup was too large to sustain intense electric fields, thus making the quadratic scenario ineffective in their experimental regime. Our model revisits and justifies this scenario in modern BPMs with nanometric junctions enabling high fields, making this mechanism not only plausible but necessary to explain observed trends.

Building on the $k_{d}$(*E*) ∝ $\;E^{2}$ result, the same quadratic law applies to the current $J\propto \;U_{j}^{2}$. For a fully hydrated, nanometric junction, this sets a clear upper-bound benchmark *J* = 660 $U_{j}$^2^. Our experiments on Fumasep^®^ FBM form a proof of principle that supports our quadratic model. The measured *J*-*U_j_* curve is already strongly quadratic before subtracting $R_{s}$ and electrode overpotentials, showing that, in reverse bias, the cell current is WD-dominated. After full junction mapping (Equation (48)), the quadratic form remains, while the fitted prefactor *K* increases; the law is robust, and reducing ohmic losses should be a priority. We attribute the remaining gap to the theoretical bound to incomplete local hydration (effective water fraction *f* < 1); improving hydration should raise *K*. Moreover, Equation (33) shows that *J* scales not only with $U_{j}$^2^ but also with 1/*δ*, inviting falsifiable tests that vary both the junction drop and the junction thickness. Finally, both theoretical and experimental results agree that WD ramps up as soon as *U_j_* > 0, with no threshold, consistent with recent observations [44,63,64]. This suggests that a strong electric field in the junction is enough to trigger WD, while interfacial catalysts are often unnecessary. Therefore, junction geometry and thickness that allow strong and uniform fields, complete hydration to maximize *f*, and parasitic-loss suppression (resistances and overpotentials) are the primary levers for pushing BPMs toward the theoretical upper bound of our power dissipation-based quadratic model. Overall, the Fumasep^®^ FBM experimental results support a quadratic, field-driven WD mechanism without additional catalysts, providing a proof of principle and motivating broader tests for validation.

Despite its simplicity, the model remains agnostic as to whether the injected power induces molecular collisions, vibrational excitation, hydrogen-bond destabilization, or solvent reorganization. Nonetheless, our approach is compatible with a thermodynamic interpretation of energy dissipation as entropy production [49]. This aligns with Chen et al. [65] and Rodellar et al. [43], who showed that high electric fields modulate interfacial entropy in BPMs via the Arrhenius pre-exponential factor. The link to high-frequency interfacial capacitance suggests that the field alters vibrational modes and molecular ordering at the junction, facilitating access to the transition state for WD. Very recently, Litman and Michaelides [66] investigated WD under strong electric fields using ab initio molecular dynamics and free-energy decomposition and showed that field-induced WD becomes predominantly entropy-driven due to protonic defects (autoprotolysis ions) disrupting an otherwise ordered hydrogen-bond network, in line with our interpretation of dissipated power and entropy production as key ingredients for field-assisted WD. Altogether, these results support a picture in which field-assisted WD in BPM junctions is sustained by local power dissipation and entropy-driven solvent reorganization. However, the precise microscopic pathways remain to be resolved via multiscale molecular dynamics (MD) simulations or in situ spectroscopy.

Finally, we acknowledge the limits and possible future directions of this research. The intricate chemistry and morphology of specific membranes may modulate the prefactor through mobilities, local activities, or effective *E_d_*; nonetheless, these variations do not undermine the functional *E*^2^ scaling. Furthermore, saturation phenomena may emerge at fields beyond the practical BPM range (*E*∼10^8^–2 × 10^9^ V·m^−1^). In such cases, it would be natural to incorporate a bounded efficiency factor or a mild saturation function in the prefactor without altering the basic *E*^2^ link. Similarly, marked dehydration or transport bottlenecks would call for couplings with water activity or layer conductivities. These are interesting directions for future work, but lie outside the intended baseline purpose of the present model.

## 6. Summary

This work introduced a power dissipation framework for electric-field-induced water dissociation (WD) in bipolar membranes, in which minority autoprotolysis ions H_3_O^+^ and OH^−^ drift under strong interfacial fields and continuously dissipate field-supplied power, thereby increasing the propensity for heterolytic O–H bond cleavage. This mechanism yields a quadratic rate law $k_{d}$(*E*) ∝ $\;E^{2}$ and an associated junction-level current law $J=K\times U_{j}^{2}$. Without adjustable parameters, the model reproduces the orders-of-magnitude enhancement $k_{d}\left(E\right)$/$k_{d}\left(0\right)\;$ across BPM-relevant fields while avoiding the numerical fragilities of exponential SWE formalisms. A proof of principle on a commercial Fumasep^®^ FBM corroborates the quadratic *J*-*U_j_* trend, supporting a field-driven, catalyst-free WD pathway under reverse bias. Altogether, the theory and experiment provide a compact, falsifiable baseline for WD in strong-field, reaction-limited, well-hydrated BPM nanometric junctions and motivate broader catalyst-free validations across materials and operating windows to establish generality.

## Figures and Tables

**Table 1:** Values of *k_d_* as a function of *E*, according to Equation (31) of our model.

Electric Field, *E* (V·m^−1^)	$\mathrm{WD}\;\mathrm{Rate},\;k_{d}$(*E*) (s^−1^)	Ratio Increase, $k_{d}\left(E\right)$$/k_{d}\left(0\right)$
1 × 10^8^	12.3	4.92 × 10^5^
5 × 10^8^	307	1.23 × 10^7^
8 × 10^8^	787	3.15 × 10^7^
1 × 10^9^	1230	4.92 × 10^7^
2 × 10^9^	4920	1.97 × 10^8^

**Table 2:** Comparison of $k_{d}\left(E\right)/k_{d}\left(0\right)$ values obtained with our model and SWE model.

Model	$k_{d}\left(E\right)/k_{d}\left(0\right)$ for *E* = 10^8^ V·m^−1^	$k_{d}\left(E\right)/k_{d}\left(0\right)$ for *E* = 2 × 10^9^ V·m^−1^	Commentary
$\mathrm{SWE}\;(\mathrm{bulk}\;\varepsilon_{r}$ = 78)	3.67	4.07 × 10^4^	Too slow
$\mathrm{SWE}\;(\varepsilon_{r}$ variable)	3.67	8.05 × 10^11^	Improved but unstable
$\mathrm{SWE}\;(\varepsilon_{r}$ = 10)	3.07 × 10^2^	3.43 × 10^15^	Divergence
$\mathrm{SWE}\;(\varepsilon_{r}$ = 5)	8.61 × 10^3^	$6.18\;\times \;10^{22}$	Extreme divergence
Our quadratic model	4.92 × 10^5^	1.97 × 10^8^	Gradual, realistic increase

## Data Availability

Data are available upon request.

## Abbreviations

$k_{d}\left(E\right)$	Water dissociation rate constant vs. field; unit s^−1^.
$\left(0\right)$	Thermal WD rate constant (no field); s^−1^.
E	Electric field in the BPM junction; V·m^−1^.
$U_{j}$	Junction voltage drop across active region; V.
δ	Junction thickness; m (e.g., 1 nm benchmark).
J	Current density at the junction; mA·cm^−2^.
K	Prefactor in junction law $J=K\;U_{j}^{2}$; mA·cm^−2^·V^−2^ (when J in mA·cm^−2^, $U_{j}$ in V).
f	Water volume fraction in the junction (hydration); dimensionless $\left[0,1\right]$.
q	Elementary charge; C (e.g., $1.6\times 10^{-19}$).
$\mu_{H_{3}O^{+}},\mu_{OH^{-}}$	Ionic mobilities; m^2^·V^−1^·s^−1^ (e.g., $3.62\times 10^{-7}$, $2.05\times 10^{-7}$ at 25 °C).
$c_{\mathrm{ions}}$	Concentration of autoprotolysis ions $H_{3}O^{+}/OH^{-}$ in water; mol·L^−1^ (≈$10^{-7}$ at 25 °C).
$c_{H_{2}O,l}$	Concentration of free water; mol·L^−1^ (≈55.5 at 25 °C).
$c_{H_{2}O,j}$	Concentration of water in the junction; mol·L^−1^
$E_{d}$	Threshold dissociation energy per water molecule; J (≈$1.33\times 10^{-19}$ at 25 °C).
$J_{\lim}$	Limiting current density (plateau); mA·cm^−2^ (≈2.19 measured here).
$E^{\backslash *}$	Crossover field where $k_{d}=k_{0}$; V·m^−1^ (≈$1.43\times 10^{5}$).
$E_{cell}$	External cell voltage applied; V.
$E_{cell}^{rev}$	Reversible (equilibrium) voltage of the cell; V (see Equation (49)).
$R_{s}$	Series resistance (two-compartment cell, area-normalized); Ω·cm^2^ (EIS-derived).
$\eta_{\mathrm{HER}},\eta_{\mathrm{OER}}$	Overpotentials for HER/OER; V.
$\Lambda^{\circ }$	Limiting molar conductivity at infinite dilution; S·m^2^·mol^−1^ (used to infer μ).
F	Faraday constant; C·mol^−1^ (96,485).
η	Viscosity (in Stokes–Einstein context for μ); Pa·s.
b	Tafel slope; mV·dec^−1^.
$j_{0}$	Exchange current density; A·cm^−2^.
$v_{d}$	Drift velocity of ions under the field; m·s^−1^. (Relation: $v_{d}=\mu E$.)
P	Per-ion field-supplied power dissipated by drift; (J·s^−1^). (Model: $P=q\;\mu \;E^{2}$.)
$P_{H_{3}O^{+}},P_{{\mathrm{OH}}^{-}}$	Per-ion power for $H_{3}O^{+}$ and ${\mathrm{OH}}^{-}$; (J·s^−1^).
$n_{H_{3}O^{+}},n_{{\mathrm{OH}}^{-}}$	Number density of autoprotolysis ions; m^−3^.
$V_{j}$	Junction volume (membrane area × active thickness δ); m^3^.
$P_{total}$	Total power dissipated in the junction by all ions; (J·s^−1^).
w	Power density dissipated by $H_{3}O^{+}/{\mathrm{OH}}^{-}$ in the junction; J·s^−1^·m^−3^.
$\Delta G_{d}$	Molar Gibbs free energy of water dissociation; (kJ·mol^−1^). (Used to compute the per-molecule threshold $E_{d}=\Delta G_{d}/N_{A}$).
$N_{A}$	Avogadro constant; mol^−1^.
κ	Thermal conductivity of water used in the heating estimate; W·m^−1^·K^−1^.
ΔT	Estimated junction temperature rise from dissipation; K.
A	Membrane area used in estimates; m^2^ (our benchmark: 1 cm^2^).
$\varepsilon_{r}$	Relative permittivity; dimensionless.
T	Absolute temperature; K (reference 298 K/25 °C).
*R*	Universal gas constant (8.314 J·mol^−1^·K^−1^).
$K_{w}$	Water equilibrium constant ($10^{-14}$ at 25 °C).
